# Clinical case report: understanding Kienböck’s disease

**DOI:** 10.1093/omcr/omaf050

**Published:** 2025-05-28

**Authors:** Ricardo A Caravantes, Daniela Saenz, Favio Reyna

**Affiliations:** Department of Medical Research, Universidad Francisco Marroquín, 6a. Calle Final, Calle Manuel F. Ayau, Zona 10, Guatemala; Department of Medical Research, Universidad Francisco Marroquín, 6a. Calle Final, Calle Manuel F. Ayau, Zona 10, Guatemala; Department of Medical Research, Universidad Francisco Marroquín, 6a. Calle Final, Calle Manuel F. Ayau, Zona 10, Guatemala

**Keywords:** Kienböck’s disease, lunate osteonecrosis, lunate bone, radiology

## Abstract

Kienböck’s disease, or lunate osteonecrosis, is a rare condition characterized by avascular necrosis of the lunate bone in the wrist. The pathophysiology involves disruption of blood supply to the lunate, leading to bone ischemia and subsequent collapse. This case report presents the clinical course and diagnostic challenges of a patient diagnosed with Kienböck’s disease. This case highlights the challenges on the importance of early diagnosis and appropriate treatment to mitigate progression and improve patient outcomes.

## Introduction

Kienböck’s disease, or lunate osteonecrosis, is a rare condition characterized by the deterioration of the lunate bone in the wrist [1]. It mainly affects young to middle-aged males involved in manual labor or repetitive wrist activities [1]. The pathophysiology involves disrupted blood supply to the lunate, leading to ischemic necrosis and bone collapse [2].

Clinically, it presents with chronic wrist pain and swelling, resulting in limited range of motion that can impair daily activities [3]. Early diagnosis is challenging due to nonspecific symptoms and the need for advanced imaging, such as MRI, to identify ischemic changes [3]. If untreated, advanced cases may cause irreversible joint damage, requiring prompt intervention to preserve wrist function and alleviate symptoms [2, 3].

This case report details a 48-year-old patient diagnosed with Kienböck’s disease, focusing on clinical manifestations and diagnostic approaches.

## Case report

A 48-year-old male presented with a two-year history of edema and pain in his right hand. He had a vertebral fracture in 2018 that resolved without complications. Initially diagnosed with tendonitis by a private physician, he underwent physical therapy and anesthetic infiltrations, which provided limited relief. Due to persistent symptoms, further evaluation was conducted.

**Figure 1:**
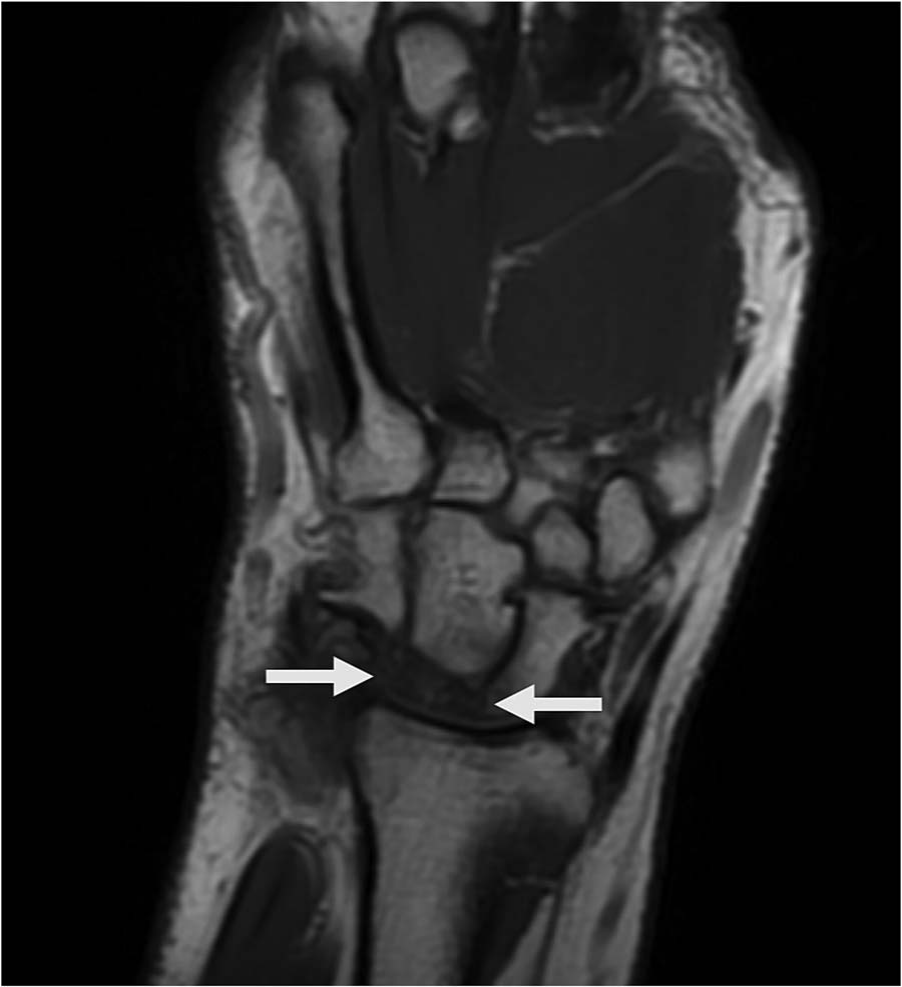
Coronal T1-weighted MR image shows marked diffuse hypointensity of lunate bone and collapse.

**Figure 2:**
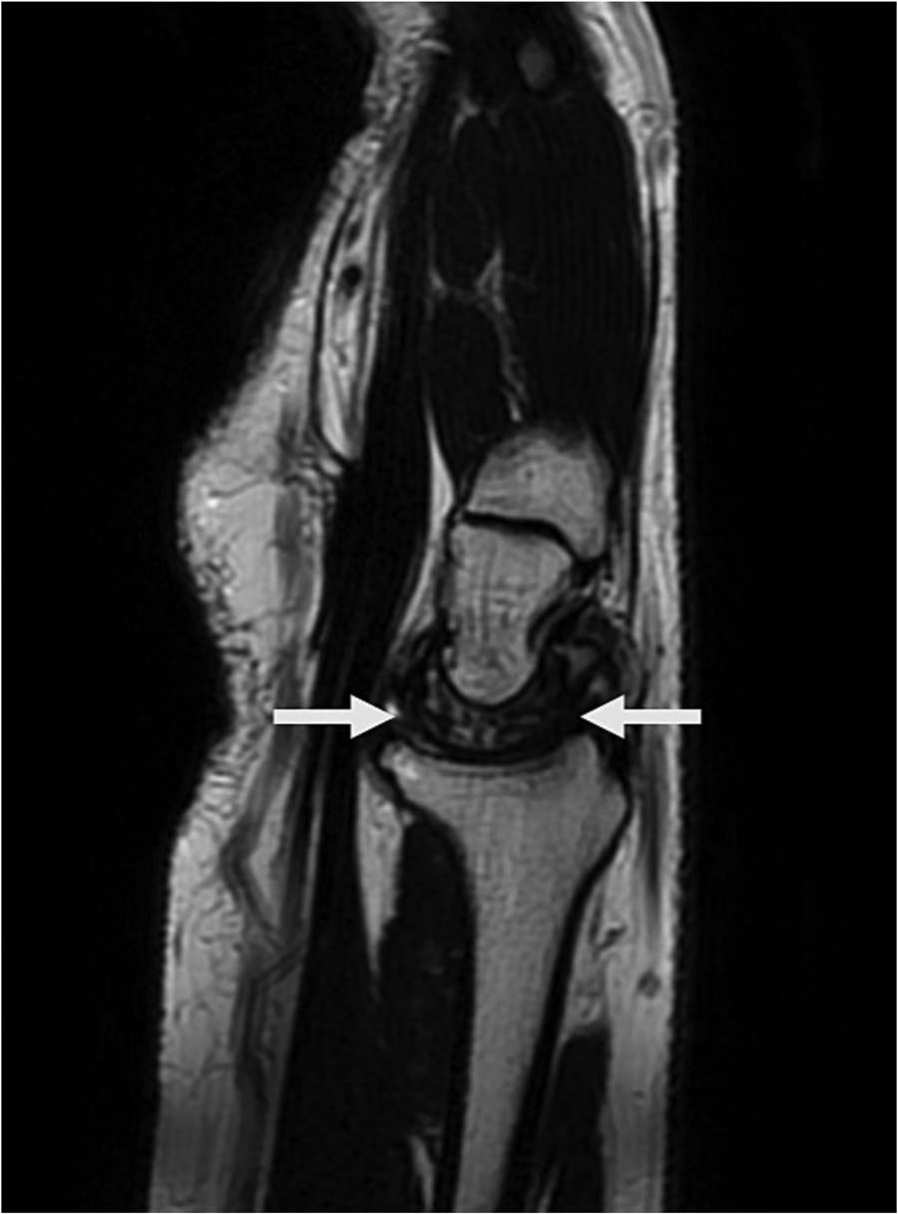
Sagittal T2 FSE image shows collapse and multifragmentary fracture of the lunate associated with sclerosis. Findings suggest chronicity.

**Figure 3:**
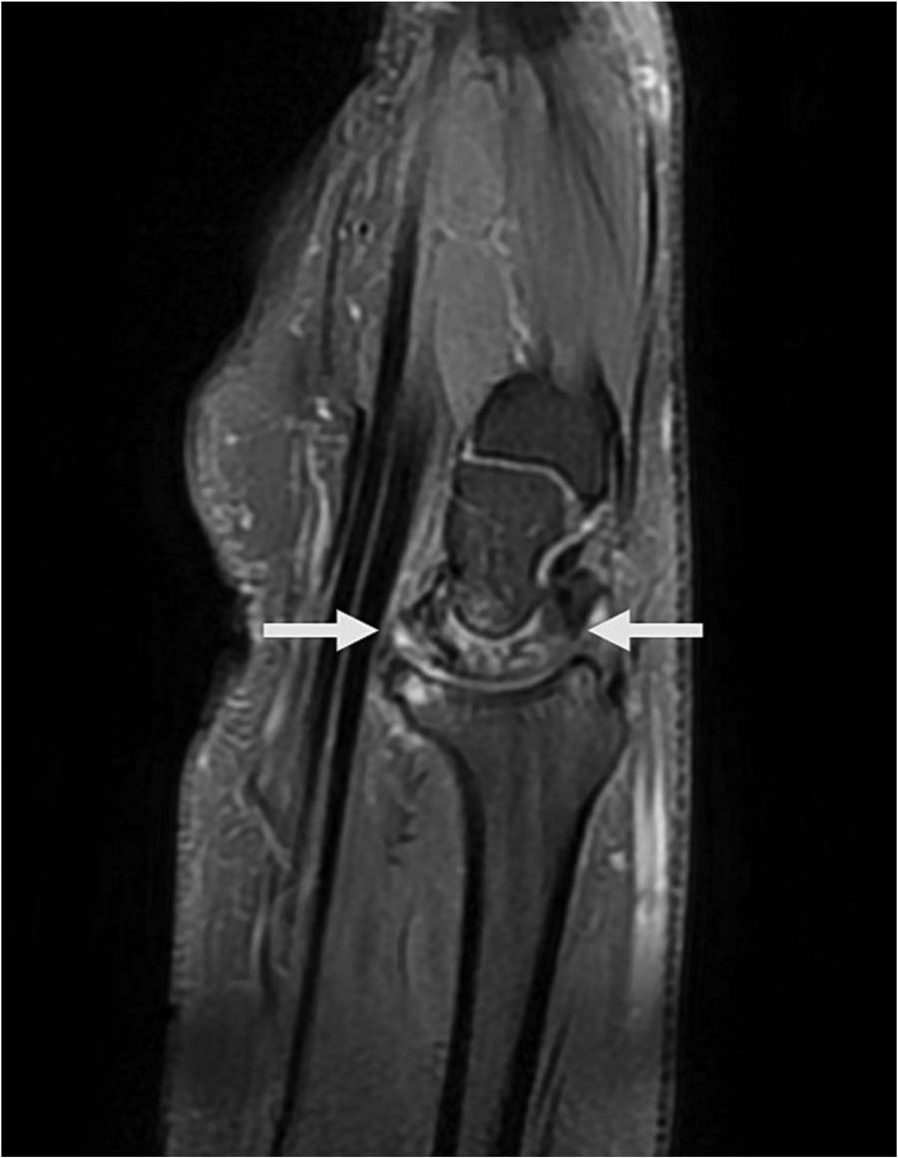
Sag PD FSE FS image shows multi-fragmentary lunate associated with arthrosis and osteoedema on both mid carpal and radio lunate articulation.

**Figure 4:**
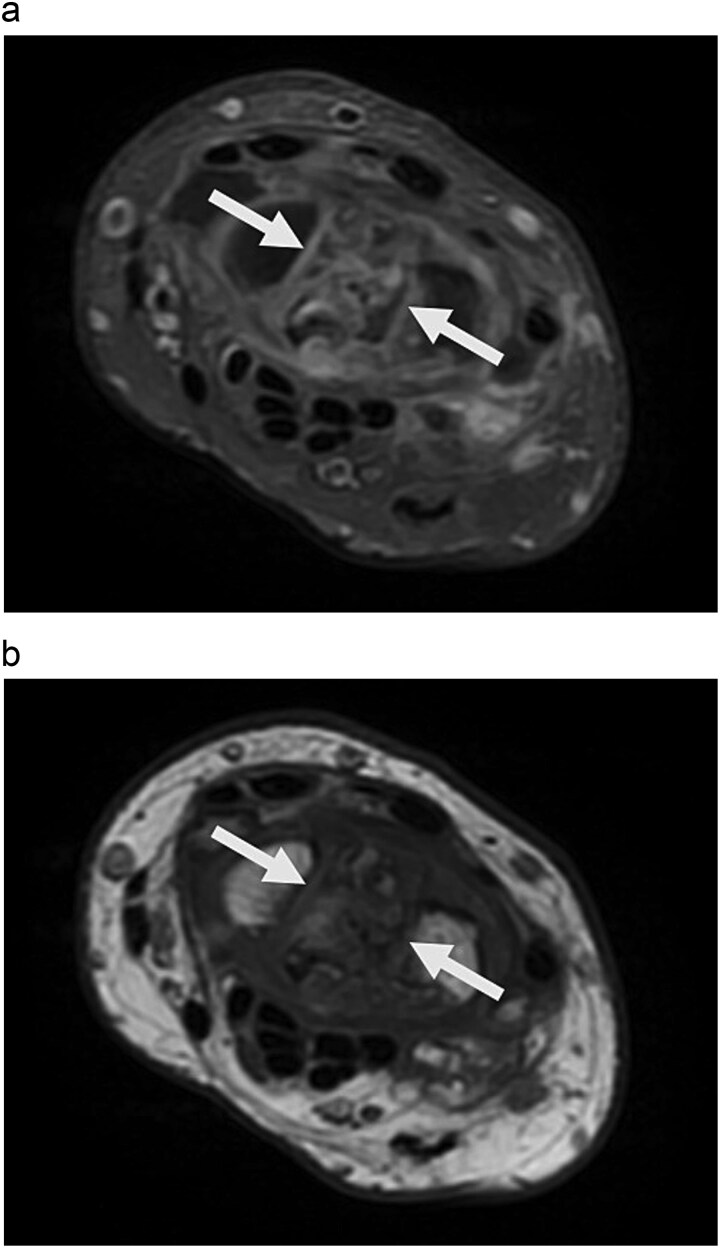
a. Axial PD FS image shows multi-fragmentary lunate associated with osteoedema. b. Axial T1 image shows diffuse, hypointense, multi-fragmentary lunate due to diffuse chronicity.

Physical examination revealed palm ecchymosis, reduced range of motion in both flexion and extension of the right wrist, and diminished grip strength. Imaging studies, including X-rays and MRI, indicated osteosynthesis of the carpal bones and fractures of the scaphoid and lunate bones. Coronal MRI showed diffuse hypointensity and collapse of the lunate (Fig. 1). Sagittal T2 FSE imaging revealed a multifragmentary fracture of the lunate with sclerosis (Figs 2 and 3). Axial PD FS images confirmed a multifragmentary lunate fracture and osteoedema (Fig. 4a and b). The patient was diagnosed with avascular necrosis of the semilunar bone of the carpus, Kienböck’s disease stage IV (according to the Lichtman classification), and carpal arthrofibrosis. A contrast-enhanced MRI was not performed, as the diagnosis of stage IV would not alter treatment.

Surgical intervention included scaphoid-lunate fusion with bone grafting, semilunar tendon arthroplasty, and radiocarpal capsulectomy. Postoperatively, the patient experienced significant improvement, though chronic pain persisted initially. He underwent a 12-session physical therapy program (three times per week), focusing on heat, stretching, shockwave therapy, and ultrasound. No pain medication was prescribed. At home, he engaged in weightlifting with 5-pound weights, used therapeutic putty, and exercised with elastic bands to enhance strength and mobility. After one year, he achieved almost full range of motion, with minimal wrist flexion restriction and normal grip strength (90%–100% of pre-injury levels). The patient expressed full satisfaction with the outcome.

## Discussion

Kienböck’s disease, or lunate osteonecrosis, is a debilitating condition primarily affecting the lunate bone in the wrist [1]. It is characterized by avascular necrosis due to interrupted blood supply, leading to bone death. This rare condition affects approximately 0.0066% of the population, predominantly men aged 20 to 40 [2, 3].

The etiology of Kienböck’s disease is largely unknown, but several contributing factors have been identified [4]. These include repetitive microtrauma, acute wrist injuries, and anatomical variations in the lunate’s blood supply, which is limited to a few vessels, making it susceptible to ischemic damage. Systemic conditions like lupus or sickle cell disease that impair blood flow may also play a role. The pathophysiology begins with an ischemic event leading to lunate necrosis, followed by changes such as bone fragmentation, collapse, and carpal instability [4].

Numerous studies have highlighted the profile of ‘at-risk’ patients, identifying genetic, anatomical, vascular, and metabolic associations. The ‘at-risk’ group predominantly consists of young, active males, particularly those engaged in manual labor, who represent over 90% of cases in some research [5].

Patients with Kienböck’s disease experience chronic wrist pain that worsens with activity and improves with rest [6]. Pain is localized to the back of the wrist, accompanied by swelling, tenderness, and reduced motion. Progression can lead to stiffness, weakness, and difficulties with daily tasks [6]. Severe cases may result in deformities and decreased grip strength, significantly impacting quality of life [6].

Diagnosing Kienböck’s disease can be challenging, especially in early stages when symptoms resemble other wrist conditions like ligament injuries or carpal instability [7]. Radiographic imaging is crucial; plain X-rays can reveal changes in the lunate, such as increased density, fragmentation, and collapse, though early-stage disease may not be visible [7]. Advances in diagnostic techniques, such as high-resolution MRI and dynamic contrast-enhanced MRI, have improved early detection of ischemic changes [8, 9]. These methods better visualize bone marrow edema and perfusion deficits, allowing for earlier intervention. Additionally, 3D CT scans enhance assessment of carpal bone morphology and alignment for surgical planning [10].

Management of Kienböck’s disease varies by stage, focusing on pain relief, wrist function preservation, and progression prevention [11]. Early stages may respond to conservative treatments like immobilization, NSAIDs, and physical therapy, but these are often ineffective in advanced stages.

When conservative treatments fail, surgical options include revascularization, joint leveling, and salvage procedures like partial or total wrist fusion [11, 12]. In stage IV, characterized by significant lunate collapse and carpal degeneration, the focus shifts to salvage procedures [13].

In this case, scaphoid-lunate intercarpal fusion was performed to stabilize the carpal bones while preserving some wrist motion [14], aiming to prevent further collapse and alleviate pain from instability.

In contrast to alternatives like proximal row carpectomy, which may relieve pain but compromise wrist strength, or total wrist arthrodesis, which offers definitive pain relief at the cost of complete motion loss, scaphoid-lunate fusion balances motion preservation with pain relief [15]. Vascularized bone grafting is less effective in stage IV due to significant lunate necrosis and carpal collapse [16]. Selecting scaphoid-lunate intercarpal fusion tailored the approach to maintain wrist function while addressing advanced disease, making it suitable for stage IV patients seeking pain relief with some mobility [14].

The prognosis of Kienböck’s disease depends on the diagnosis stage and treatment effectiveness. Early intervention can improve outcomes, with many patients experiencing pain relief and functional gains [9]. However, advanced stages may lead to ongoing pain and functional impairment despite surgery. Long-term follow-up is crucial to monitor disease progression and manage complications [9].

## Conclusion

Kienböck’s disease involves avascular necrosis of the lunate bone in the wrist, primarily affecting young to middle-aged adults. This case discusses a patient with advanced Kienböck’s disease, presenting chronic wrist pain, reduced range of motion, and functional impairment. Management requires a tailored approach based on disease stage and individual factors. Early diagnosis is crucial for effective treatment to preserve wrist function and slow disease progression. Ongoing research and advancements in imaging will enhance our understanding and management of this condition. Long-term outcomes highlight the need for multidisciplinary collaboration and personalized treatment plans to improve patient outcomes and quality of life.
